# Characterization of Circulating T Cell Receptor Repertoire Provides Information about Clinical Outcome after PD-1 Blockade in Advanced Non-Small Cell Lung Cancer Patients

**DOI:** 10.3390/cancers13122950

**Published:** 2021-06-12

**Authors:** Ning Dong, Andrea Moreno-Manuel, Silvia Calabuig-Fariñas, Sandra Gallach, Feiyu Zhang, Ana Blasco, Francisco Aparisi, Marina Meri-Abad, Ricardo Guijarro, Rafael Sirera, Carlos Camps, Eloísa Jantus-Lewintre

**Affiliations:** 1 Molecular Oncology Laboratory, Fundación Investigación, Hospital General Universitario de Valencia, 46014 Valencia, Spain; dongning@alumni.uv.es (N.D.); moreno_andman@externos.gva.es (A.M.-M.); calabuix_sil@gva.es (S.C.-F.); gallach_sangar@gva.es (S.G.); zfei@alumni.uv.es (F.Z.); 2 Unidad Mixta TRIAL, Centro Investigación Príncipe Felipe—Fundación Investigación, Hospital General Universitario de Valencia, 46014 Valencia, Spain; blasco_ana@gva.es (A.B.); guijarro_ricjor@gva.es (R.G.); rsirera@btc.upv.es (R.S.); 3 Centro de Investigación Biomédica en Red Cáncer, CIBERONC, 28029 Madrid, Spain; 4 Department of Pathology, Universitat de València, 46010 Valencia, Spain; 5 Department of Medical Oncology, Hospital General Universitario de Valencia, 46014 Valencia, Spain; mamea2@alumni.uv.es; 6 Department of Medical Oncology, Hospital General de Requena, 46340 Valencia, Spain; aparisi_fraapa@gva.es; 7 Department of Surgery, Universitat de València, 46010 Valencia, Spain; 8 Department of Thoracic Surgery, Hospital General Universitario de Valencia, 46014 Valencia, Spain; 9 Department of Biotechnology, Universitat Politècnica de València, 46022 Valencia, Spain; 10 Department of Medicine, Universitat de València, 46010 Valencia, Spain

**Keywords:** non-small cell lung cancer, immune checkpoint blockade, immunotherapy, biomarker, T cell receptor beta chain repertoire, high-throughput sequencing, liquid biopsy, next-generation sequencing, TCR, CDR3

## Abstract

**Simple Summary:**

Immune checkpoint blockers (ICBs) have demonstrated durable anti-tumor responses in advanced non-small cell lung cancer (NSCLC). Despite progress in development of new predictive biomarkers, such as PD-L1 expression, TMB, or MSI, there is still an urge for a better selection of patients that will benefit from the blockade of PD-1/PD-L1 axis. In this study, peripheral blood T cell receptor beta chain (TCR-β) repertoire, at baseline (PRE) and first response (FR) assessment, was analyzed with high-throughput sequencing in a cohort of advanced NSCLC patients receiving first-line pembrolizumab. Our results suggest that measuring TCR-β features in peripheral blood may be a potential tool to assess patients’ immune response. Furthermore, the usage of the TRBV20-1 segment highly predicts host response and survival in anti-PD-1 treated NSCLC patients.

**Abstract:**

Despite the success of immunotherapies in lung cancer, development of new biomarkers for patient selection is urgently needed. This study aims to explore minimally invasive approaches to characterize circulating T cell receptor beta chain (TCR-β) repertoire in a cohort of advanced non-small cell lung cancer (NSCLC) patients treated with first-line pembrolizumab. Peripheral blood samples were obtained at two time points: i) pretreatment (PRE) and ii) first response assessment (FR). Next-generation sequencing (NGS) was used to analyze the hypervariable complementary determining region 3 (CDR3) of TCR-β chain. Richness, evenness, convergence, and Jaccard similarity indexes plus variable (V) and joining (J)-gene usage were studied. Our results revealed that increased richness during treatment was associated with durable clinical benefit (DCB; *p* = 0.046), longer progression-free survival (PFS; *p* = 0.007) and overall survival (OS; *p* = 0.05). Patients with Jaccard similarity index ≥0.0605 between PRE and FR samples showed improved PFS (*p* = 0.021). Higher TRBV20-1 PRE usage was associated with DCB (*p* = 0.027). TRBV20-1 levels ≥9.14% in PRE and ≥9.02% in FR significantly increased PFS (*p* = 0.025 and *p* = 0.016) and OS (*p* = 0.035 and *p* = 0.018). Overall, analysis of circulating TCR-β repertoire may provide information about the immune response in anti-PD-1 treated NSCLC patients; in this scenario, it can also offer important information about the clinical outcome.

## 1. Introduction

Lung cancer is one of the leading causes of cancer death worldwide, with a 5-year overall survival rate of less than 20% [1,2]. Recent advancements in immunotherapy, focused on immune checkpoint blockers (ICBs), specifically programmed cell death protein 1 (PD-1) and programmed death-ligand 1 (PD-L1) inhibitors in different histologic features, have opened up new horizons and remarkably improved the clinical outcomes of patients with non-small cell lung cancer (NSCLC) [3,4,5]. At present, the expression level of PD-L1 on tumor cells and/or tumor-infiltrating immune cells has been correlated with the response to ICBs and is considered the most available and implemented biomarker to select patients [6]. However, there is evidence that many patients whose tumor cells express high levels of PD-L1 exhibit poor response, while others with low or undetectable levels of PD-L1 show objective responses to ICBs, limiting its use in routine clinical practice [7,8].

Recently, additional biomarkers, such as microsatellite instability-high/deficient mismatch repair (MSI-H/dMMR) and tumor mutational burden (TMB), have been approved by the U.S. Food and Drug Administration (FDA) as additional ICB predictive biomarkers [9,10,11]. Multiple data have supported that high TMB is associated with improved response to ICBs therapy across several cancer types, including NSCLC [12,13,14]. Nevertheless, some limitations, such as the absence of standardization of TMB according to tumor histologies, no consistent correlation with overall survival, and the evidence that this biomarker does not correlate properly and linearly with the neoantigen load, highlight that the predictive value of TMB for immunotherapy must be confirmed in more precise studies [15]. Furthermore, one additional challenge for TMB estimation is the lack of adequate tissue available at diagnosis. In this way, a pilot study has shown the feasibility of applying TMB determination to cytological samples, although this finding needs to be confirmed in more extensive cohorts [16]. Therefore, new predictive and prognostic biomarkers are still under study to accurately direct therapeutic decisions. Among others, T cell profiling may have great relevance since it is closely related to the presence of tumor neoantigens.

**Figure 1:**
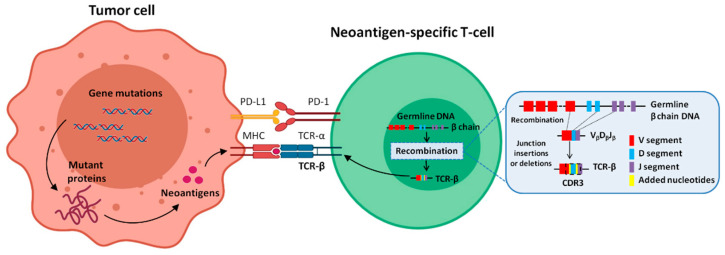
Relationship between tumor cell and neoantigen-specific T-cell. On one hand, tumor cells with high tumor mutational burden (TMB) produce an elevated neoantigen load. The neoantigens are processed within the tumor cell, presented by the major histocompatibility complex (MHC) molecule, and recognized by a neoantigen-specific T cell receptor (TCR). On the other hand, the TCR is represented on the T cell surface, consisting of a heterodimeric protein comprising two chains: α (TCR-α) and β (TCR-β). The highly variable complementarity determining region 3 (CDR3) of TCR-β is encoded at the gene level, in the variable (V), diversity (D), and joining (J) segments. During somatic diversification, one of each segment is randomly combined, and further diversity is introduced through insertions and/or deletions of added nucleotides. CDR3 is the main region responsible for recognizing processed antigens.

Tumors with high TMB, such as melanoma and NSCLC, present an elevated production of neoantigens, which are mutant proteins generated by a variety of non-synonymous genetic alterations of tumor cell genomes [14,17]. When processed within the tumor cell and presented by the major histocompatibility complex (MHC) molecules, neoantigens can be captured and recognized by specific T cell receptors (TCR) via TCR/peptide/MHC interactions [18] (Figure 1). These neoantigen-specific T cell clones can be further reactivated by monoclonal antibodies to avoid immune escape and reinforce the host anti-tumor immune response [19]. Accordingly, TCR repertoire study allows a more accurate approach and opens a new possibility for a more selective and effective biomarker assessment.

In αβ T cells, most of the TCR diversity is contained within the third complementarity-determining region (CDR3) of the α (TCR-α) and β (TCR-β) chains, the TCR-β being more diverse than the TCR-α [20]. The specificity and diversity of CDR3 are generated by random recombination of the variable (V), diversity (D), and joining (J) gene segments (Figure 1) [21]. When a T cell is activated and clonally expanded, all the cells of the clonal lineage express the same CDR3 [22]. Nonetheless, limited reports correlating TCR-β profiling response of immunotherapy have been published and, thereby, more comprehensive studies are required to confirm TCR-β diversity as an immuno-oncology biomarker.

More recently, the development of next-generation sequencing (NGS) methods has facilitated the characterization of TCR-β repertoire in different solid tumor types [23]. Nonetheless, in NSCLC, the knowledge of the impact of the immune repertoire in the context of immune-based therapies was focused on the TCR sequencing in tumor-infiltrating lymphocytes (TILs) [24,25,26,27]. Intratumoral heterogeneity has been observed in TCR clonality of CD4+ and CD8+ TILs across different regions of the same tumor, which may be the result of spatial and temporal diversity of neoantigens during lung cancer evolution [28,29,30]. Therefore, in order to dynamically track changes in tumor immune environment, alternative approaches are required. Liquid biopsy based on minimally invasive samples, such as peripheral blood, is a potential tool to solve the lack of tumor, and to better recapitulate tumor heterogeneity and plasticity. In addition, this approach with low failure rate usually requires short processing time and is less expensive [31]. Moreover, it has been proposed that the blood-based biomarkers, such as absolute lymphocyte count (ALC), neutrophil–lymphocyte ratio (NLR), etc., can show prognostic and predictive significance in lung cancer patients, but not prospectively validated [32,33]. In this regard, we think that an in-depth analysis of the T cell repertoire in the peripheral blood could be a rich source to unravel the dynamic behavior of the immune compartment during the course of immunotherapy.

In this study, we investigated the TCR-β repertoire at pretreatment (PRE) and first response assessment (FR) in peripheral blood mononuclear cells (PBMCs) to predict clinical outcomes in advanced NSCLC patients treated with first-line pembrolizumab by means of a minimally invasive approach. Our results further support that sequencing of circulating TCR-β CDR3 may be used as a biomarker of response and the presence and frequency of certain clonotypes might predict increased survival to anti-PD-1 therapy.

## 2. Materials and Methods

### 2.1. Patients

Between May 2019 and September 2020, a total of 33 advanced NSCLC patients treated with first-line pembrolizumab in monotherapy (200 mg every 21 days; *n* = 24) or in combination with chemotherapy (pembrolizumab 200 mg, cisplatin 130 mg, pemetrexed 200 mg, every 21 days; *n* = 9) were enrolled in this study. Patients with autoimmune disease or AIDs were excluded from the study. Demographic and clinicopathological characteristics for all patients were collected. Follow-up was performed according to the institutional standard for advanced NSCLC patients treated with ICB.

The study was conducted in accordance with the Declaration of Helsinki, and the protocol was approved by the ethical review board of the General University Hospital of Valencia. All patients provided written informed consent for sample acquisition for research purposes.

### 2.2. Sample Collection and Processing

Peripheral blood was collected in EDTA-containing tubes at baseline (PRE), prior to the first administration of pembrolizumab for all patients. One additional blood sample from 19 patients treated with pembrolizumab monotherapy was obtained at first response assessment (FR). Peripheral blood mononuclear cells (PBMCs) were isolated from whole blood using Ficoll-Paque Plus (GE Healthcare, Uppsala, Sweden) and stored in RNAlater (Thermo Fisher Scientific, Waltham, MA, USA) at −80 °C until further processing. Total RNA was isolated from PBMCs using the MagMAX mirVana Total RNA Isolation Kit (Thermo Fisher Scientific) and quantified through the Qubit RNA HS Assay Kit (Thermo Fisher Scientific). The Agilent RNA 6000 Pico Assay (Agilent Technologies, Palo Alto, CA, USA) was used to determine RNA integrity numbers (RIN) in the Agilent 2100 Bioanalyzer. A RIN value greater or equal to 7 is recommended to follow subsequent TCR-β sequencing.

### 2.3. Library Preparation and TCR-β Sequencing

Complementary DNA (cDNA) was synthesized from 25 ng of isolated RNA, using the SuperScript VILO cDNA Synthesis kit (Thermo Fisher Scientific). TCR-β CDR3 libraries were prepared from 25 ng cDNA of each sample with the Oncomine TCR Beta-SR (RNA) Assays (Thermo Fisher Scientific) according to the manufacturer’s instructions. Eight libraries of 25 pM were combined and multiplexed per Ion 530^TM^ Chip using the Ion Chef^TM^ instrument and sequenced on Ion GeneStudio S5^TM^ Series (Thermo Fisher Scientific). Matched PRE and FR samples were always sequenced in the same run.

### 2.4. Sequencing Analysis

More than 1 million raw sequencing reads were obtained with a length of around 80 base pairs (bp). After filtering the non-productive and off-target or low-quality reads, 50% or more total productive reads (productive and rescued productive) were used to identify the TCR-β CDR3 clones, according to the ImMunoGeneTics (IMGT) V, D, and J-gene reference using Ion Reporter software version 5.10 (Thermo Fisher Scientific).

T cell repertoire metrics were reported using several pre-calculated outputs, including richness, evenness, convergence, and V-gene (TRBV) and J-gene (TRBJ) usage frequencies. Richness was determined as the number of unique TCR-β CDR3 sequences [34]. Evenness, also known as the normalized diversity, was calculated as the following Equation (1):

(1) $$\frac{-\sum_{i=1}^{R}p_{i}\log_{2}\left(p_{i}\right)}{\log_{2}\left(R\right)},$$

where *p_i_* indicates the frequency of the *i^th^* clone and *R* indicates the total number of clones. Evenness accounted for both richness and the distribution inequality of the TCR-β CDR3 sequences present in each sample. Evenness values ranged from 0 to 1; the lower the evenness, the more uneven or more clonality the TCR clones distribution [35]. Evenness is the inverse of clonality (calculated as 1-evenness), thus lower evenness typically means higher clonality [36]. TCR convergence was defined as the aggregate frequency of clonotypes that are identical in amino acid space but different in nucleotide space [37]. A change in each index was defined as the logarithm of the index ratio between the two time points in base 10 (log_10_ (FR/PRE)). A log_10_ (FR/PRE) greater than 0 represents the increased index from PRE to FR.

TCR-β repertoire similarity between PRE and FR samples was determined using the Jaccard index, which was calculated as the total number of shared clones divided by the total number of unique clones across two samples; this index ranged from 0 to 1, with 0 and 1 representing minimal and maximal similarity, respectively [38].

### 2.5. Therapeutic Responses Evaluation

The objective response rate (ORR) was evaluated using the Response Evaluation Criteria in Solid Tumors version 1.1 (RECIST 1.1) [39] and was defined as the proportion of patients achieving complete (CR) or partial response (PR), stable disease (SD), and progressive disease (PD). The RECIST 1.1 quantification of response was used to assign patient designation as durable clinical benefit (DCB; CR, PR, or SD lasting over 6 months) and non-DCB (PD within 6 months after treatment start).

Progression-free survival (PFS) and overall survival (OS) were defined as the interval from the start of anti-PD-1 treatment to the endpoint (objective disease progression and death, respectively) or last follow-up.

### 2.6. Statistical Analysis

In order to compare paired PRE and FR samples, the Wilcoxon signed-rank test was used. Comparisons between any two groups were analyzed using the Mann–Whitney test and Fisher’s exact test for continuous and categorical variables, respectively. Graphs comparing metrics across groups show the median and the interquartile range, assuming non-normally distributed data. Correlation between continuous variables was assessed by the Spearman rank correlation test. 

The Kaplan–Meier method was used to calculate survival rates (PFS and OS). Log-rank tests were used for comparisons between the two groups. Hazard ratio (HR) and 95% confidence interval (95%CI) were estimated using the univariate Cox proportional hazards regression model. 

All statistical analyses were performed using the Statistical Package for the Social Sciences (SPSS) software version 24.0 (IBM Corporation, Armonk, NY, USA) and GraphPad Prism 9 software (GraphPad Software, La Jolla, CA, USA). Two-sided *p*-values < 0.05 were considered statistically significant.

## 3. Results

### 3.1. Patient Clinicopathological Characteristics

Clinical and pathological information including age, gender, stage of disease, histology, PD-L1 expression, and treatment scheme of the study cohort are summarized in Table 1. The majority of the patients in this cohort were smokers (75.8%) and males (72.7%). None of the patients harbored targetable drivers approved by European Medicines Agency (EMA). According to current guidelines, PD-L1 expression ≥50% was present in tumor samples from the 24 patients treated with pembrolizumab in monotherapy, while six patients treated with pembrolizumab plus chemotherapy presented PD-L1 expression lower than 1%. 

The ORR was 36.4% (12/33), 22 patients presented DCB (12 PR and 10 SD), whereas the remaining 11 patients non-DCB. The median duration of follow-up was 12.7 months (range: 3.1–21.7 months). Detailed patient characteristics and outcomes are shown in Table S1.

### 3.2. Sequencing Data

RNA was isolated from a total of 52 PBMCs samples, the median value of the RNA integrity number (RIN) obtained was 9.3 (7.0–10.0), thus all the samples were considered of good quality to proceed with library preparation and sequencing.

Sequencing data across the CDR3 region of TCR-β of the 52 libraries resulted in a mean of 1,431,317 (552,776–2,427,120) total productive reads (productive plus rescued reads) per sample, with an average length of 86 base pairs. Only PRE and FR samples with ≥ 50% total productive reads were used for TCR data analysis. No difference in the total number of reads between PRE and FR samples was observed (*p* = 0.376; Table S3). Detailed information of sequencing metrics for each sample is provided in Figure S1 and Table S2.

Next, we evaluated the characteristics of the TCR-β repertoire by means of different indexes, such as: richness (determines the number of unique TCR-β CDR3 sequences), evenness (measurement of the similarity of clone sizes), convergence (frequency of clonotypes that are identical in amino acid space, but different in nucleotide space), and Jaccard similarity index (ratio of the intersection to the union of the set of clones in each pair of PRE and FR samples) (see detailed index information in Table S2). Similar richness (*p* = 0.658), evenness (*p* = 0.809), and convergence (*p* = 0.107) of the TCR-β repertoire were observed between PRE and FR samples (Table S3).

### 3.3. Association of TCR-β Repertoire with Clinicopathological Characteristics

**Figure 2:**
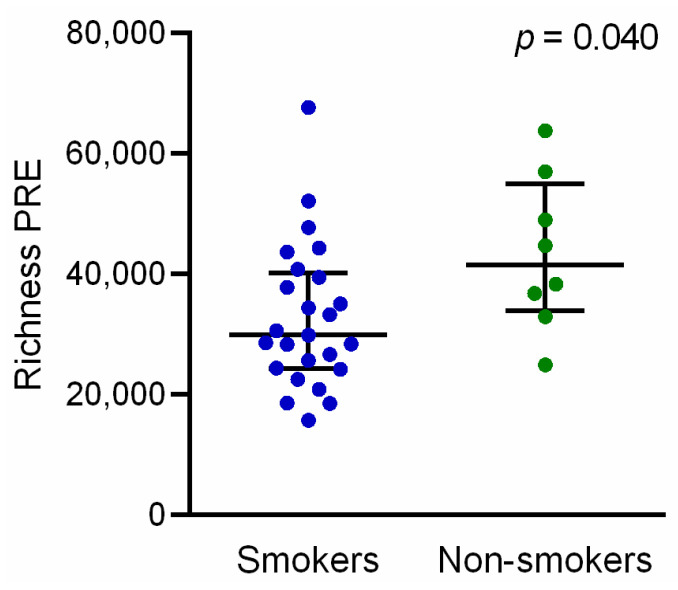
Peripheral TCR-β clone richness stratified by smoking status in pretreated NSCLC patients. *P*-value was obtained using the Mann–Whitney test. Error bars represent the interquartile range with a line at the median. Each dot represents a patient. Non-smokers: never smokers + former smokers. PRE: pretreatment.

To assess the influence of the patients’ clinicopathological characteristics on the TCR-β repertoire, we performed correlations between PRE indexes and the clinical variables. Of note, TCR-β richness was significantly lower (*p* = 0.040) in smokers compared with non-smokers (including former and never smokers) (Figure 2). No significant associations were found with the rest of the clinicopathological characteristics (age, histology, stage of disease, tumor size, number of metastatic localizations, and PD-L1 expression) analyzed at baseline (Table S4).

### 3.4. Dynamic Monitoring of TCR-β Repertoire during Immunotherapy Treatment Provides Prognostic and Predictive Information

We further investigated the correlation of PRE TCR-β repertoire characteristics with clinical outcomes to anti-PD-1 treatment and no significant associations were observed (Figures S2 and S3). However, we found a trend toward longer PFS and OS among patients with lower richness and higher evenness at baseline (Figure S3).

**Figure 3:**
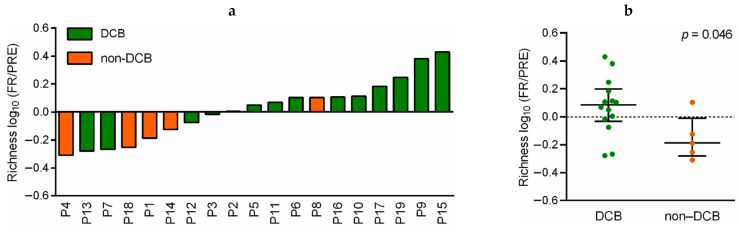
Correlation of dynamic richness of TCR-β repertoire in peripheral blood with response in anti-PD-1 treated NSCLC patients. (**a**) Richness log_10_ (FR/PRE) between pretreatment (PRE) and first response assessment (FR) in patients with durable clinical benefit (DCB; in green) and non-durable clinical benefit (non-DCB; in orange). Each column represents a patient. (**b**) Comparison of richness log_10_ (FR/PRE) between patients with DCB and non-DCB. *P*-value was obtained using the Mann–Whitney test. Error bars represent the interquartile range with a line at the median. Each dot represents a patient.

**Figure 4:**
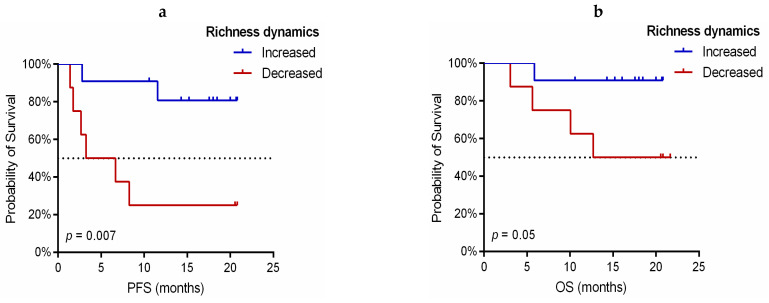
Kaplan–Meier survival curves according to TCR-β richness dynamics in anti-PD-1 treated NSCLC patients. (**a**) Progression-free survival (PFS) stratified in increased (*n* = 11) vs. decreased richness (*n* = 8). （**b**） Overall survival (OS) stratified in increased (*n* = 11) vs. decreased richness (*n* = 8). *P*-values were obtained using the log-rank test.

Additionally, the changes in the TCR-β repertoire of 19 paired PRE and FR collected samples from patients treated with pembrolizumab monotherapy were analyzed and correlated with clinical outcomes. Regarding the dynamics of richness index during treatment, an increase in this parameter was found in 10 patients with DCB (10/14, 71.4%) and only in one non-DCB patient (P8) (1/5, 20%; Figure 3a). Moreover, the number of unique TCR clones was significantly increased after the introduction of anti-PD-1 therapy in patients with DCB versus non-DCB (*p* = 0.046; Figure 3b and Table S5). In this line, we found that patients with increased TCR richness (FR compared to PRE) showed improved PFS (HR: 0.143, 95%CI: 0.028–0.727, log-rank *p* = 0.007; Figure 4a) and OS (HR: 0.152, 95%CI: 0.017–1.361, log-rank *p* = 0.05; Figure 4b).

Considering these observations, we questioned if the TCR richness increase was linked to the dynamics of the absolute lymphocytes count (ALC) in circulation. Among 11 patients whose richness increased during treatment, we identified ix patients (P2, P8, P9, P11, P16, and P17) with an increase of ALC; nevertheless, the other five patients (P5, P6, P10, P15, and P19) presented a decrease in ALC. The correlation analysis showed no significant correlation between ALC and TCR richness changes (Figure S4).

Peripheral TCR-β evenness changes were small in magnitude during immunotherapy and no dynamic pattern in terms of the TCR convergence was observed in NSCLC patients’ cohort (Figures S5 and S6). However, the three patients (P2, P9, and P15) who showed a dramatic increase of convergence (log_10_ (FR/PRE) = 0.41, 0.28, and 0.53, respectively) exhibited PR to ICB therapy.

### 3.5. Association of TCR-β Clones Similarity before and during Immunotherapy Treatment with Predictive and Prognostic Variables

**Figure 5:**
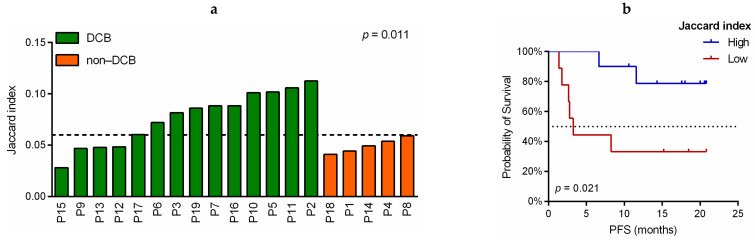
Correlation of TCR-β similarities before and during the anti-PD-1 treatment with response and prognostic variables in NSCLC patients. (**a**) Jaccard index in patients with durable clinical benefit (DCB; in green) and non-durable clinical benefit (non-DCB; in orange). *p*-value was obtained using the Fisher’s exact test. Each column represents a patient. (**b**) Kaplan–Meier progression-free survival (PFS) curves according to the Jaccard similarity index. Jaccard similarity index was calculated as the total number of shared clones divided by the total number of unique clones across PRE and FR samples of the same patient and the median = 0.0605 was used as the cutoff value to define high (blue line) and low (red line) groups. *p*-value was obtained using the log-rank test.

The Jaccard index was used to evaluate the similarity of CDR3 sequences between PRE and FR samples for 19 patients treated with pembrolizumab. Compared to non-DCB patients, patients who exhibited DCB showed a trend towards increased Jaccard similarity, i.e., greater proportion of shared CDR3 sequences between PRE and FR samples (*p* = 0.052; Figure S7 and Table S5). Interestingly, 10 of 14 (71.4%) patients who exhibited DCB presented a Jaccard index greater or equal to the median (median = 0.0605, defined as “high Jaccard”), whereas none of the non-DCB patients showed high Jaccard index (*p* = 0.011) (Figure 5a). Furthermore, PFS was significantly longer in patients with high Jaccard index (HR: 0.183, 95%CI: 0.037–0.921, log-rank *p* = 0.021; Figure 5b) and a similar trend was observed in OS (HR: 0.173, 95%CI: 0.019–1.559, log-rank *p* = 0.077; Figure S8).

### 3.6. Implication of V and J-Gene of Peripheral TCR-β Repertoire

**Figure 6:**
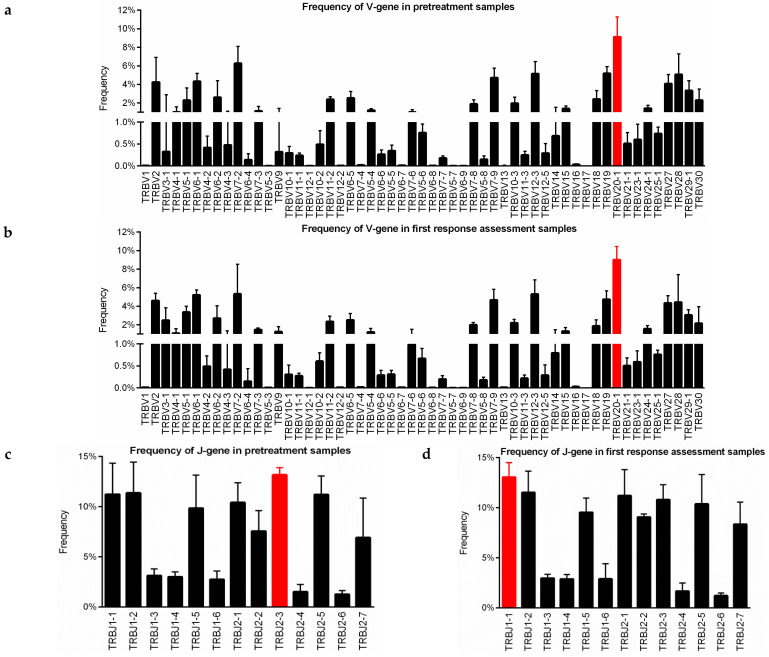
V and J-gene usage in anti-PD-1 treated NSCLC patients. (**a**,**b**) Frequency of V-gene in pretreatment and first response assessment samples. (**c**,**d**) Frequency of J-gene in pretreatment and first response assessment samples. The height of each column represents the median of the frequency and the error bars depict the interquartile range. The red columns correspond to V and J segments with the highest frequencies for pretreatment and first response assessment samples.

The V and J-gene usage was analyzed in PRE and FR peripheral blood samples to investigate the distribution of the V and J segments, aside from the correlation of dominant use of certain segments with follow-up. The NGS assay detected 55 distinct V-gene segments and 13 distinct J-gene segments. Similar TRBV and TRBJ gene usage of the TCR-β repertoire in 33 PRE and 19 FR samples was observed through heatmap analyses (Figure S9). Based on the frequencies of TRBV usage, the top three most abundant V segments in PRE samples were TRBV20-1 (9.14%), TRBV7-2 (6.28%), and TRBV19 (5.19%) (Figure 6a). While TRBV20-1 (9.02%), TRBV7-2 (5.33%), and TRBV12-3 (5.31%) presented the highest frequencies at first response assessment (Figure 6b). Among J-gene segments, TRBJ2-3 was the most frequent in PRE (13.17%; Figure 6c) and TRBJ1-1 in FR samples (13.05%; Figure 6d).

**Figure 7:**
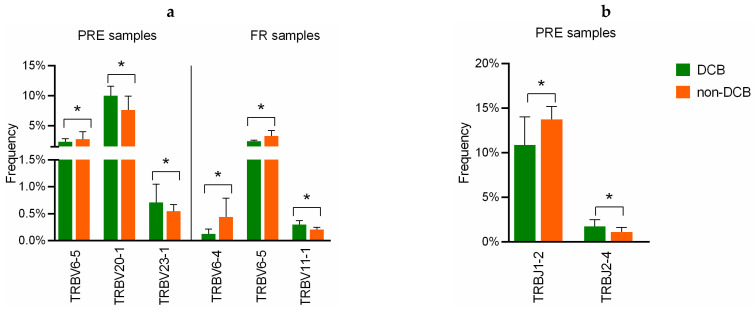
Comparison of V and J-gene usage between anti-PD-1 treated NSCLC patients with DCB vs. non-DCB. (**a**) V-gene usage in pretreatment (PRE) and first response assessment (FR) samples. (**b**) J-gene usage in PRE samples. Statistical analysis was performed using the Mann–Whitney test. The height of each column represents the median of the frequency and the error bars depict the interquartile range. DCB: durable clinical benefit, in green; non-DCB: non-durable clinical benefit, in orange. * *p* < 0.05.

Afterwards, we compared the frequency of clonotypes between DCB and non-DCB patients (Table S5). Specifically, TRBV20-1, as the most abundant segment V, was more abundant in DCB patients at baseline (*p* = 0.027; Figure 7a). Among others V segments with relatively low usage, baseline TRBV23-1 was more frequent in patients with DCB (*p* = 0.043) and TRBV6-5 was more frequent in non-DCB (*p* = 0.013). In the case of FR samples, patients with DCB showed higher frequency of TRBV11-1 (*p* = 0.033), while patients with non-DCB showed higher frequencies of TRBV6-4 (*p* = 0.033) and TRBV6-5 (*p* = 0.010) (Figure 7a). According to J segments usage, TRBJ1-2 PRE was more enriched in non-DCB (*p* = 0.047), whereas TRBV2-4 PRE was significantly enriched in patients with DCB (*p* = 0.032) (Figure 7b). In FR samples, no significant differences in the usage of TRBJ segments were observed.

**Figure 8:**
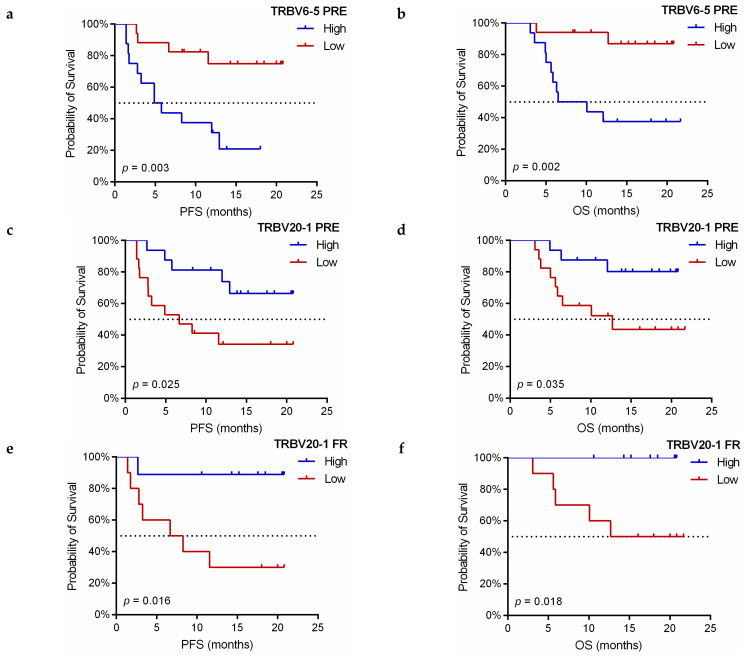
Kaplan–Meier survival curves according to V-gene usage in anti-PD-1 treated NSCLC patients. (**a**,**b**) Progression-free survival (PFS) and overall survival (OS) curves in terms of PRE TRBV6-5 frequency. (**c**,**d**) PFS and OS curves in terms of PRE TRBV20-1 frequency. (**e**,**f**) PFS and OS curves in terms of FR TRBV20-1 frequency. The median frequency values were used as cutoff values (2.53% for PRE TRBV6-5, 9.14% for PRE TRBV20-1, and 9.02% for FR TRBV20-1, respectively). PRE: pretreatment, FR: first response assessment, *p*-values were obtained using the log-rank test.

When categorizing patients as having higher (≥ median) or lower (< median) frequencies of TRBV segments usage, TRBV6-5 and TRBV20-1 showed a significant correlation with PFS and OS. High TRBV6-5 PRE was associated with a worse PFS (HR: 4.770, 95%CI: 1.524–14.928, log-rank *p* = 0.003; Figure 8a) and OS (HR: 7.678, 95%CI: 1.662–35.458, log-rank *p* = 0.002; Figure 8b), whereas patients with high TRBV20-1 at baseline showed a significant increase in PFS (HR: 0.314, 95%CI: 0.108–0.914, log-rank *p* = 0.025; Figure 8c) and OS (HR: 0.268, 95%CI: 0.072–0.995, log-rank *p* = 0.035; Figure 8d).

It was observed that all patients with high TRBV20-1 PRE maintained this status without dramatic changes at the time of FR assessment. When analyzing the prognostic significance of TRBV20-1 in FR samples, its high usage was also positively correlated with improved PFS (HR: 0.117, 95%CI: 0.014-0.955, log-rank *p* = 0.016; Figure 8e) and OS (HR: 0.119, 95%CI: 0.020-0.692, log-rank *p* = 0.018; Figure 8f).

## 4. Discussion

Despite the success of ICBs based therapies in NSCLC, there is a need for the development of new biomarkers for patient selection. Several tumor-based markers, such as PD-L1 expression, TMB, or MSI, have been correlated with the response to anti-PD-1 antibodies; however, there is still an urge for better identification of patients more likely to respond.

There is evidence that the analysis of TCR profiles provides predictive information of response to ICB [23]. Previous studies have analyzed the TCR-β repertoire at tumor microenvironment, showing that higher intratumoral clonality and the peripheral expansion of dominant tumor-resident T cell clones following ICB therapy are associated with better clinical outcomes [24,25,26,27]. However, in a high percentage of advanced lung cancer patients, tumor samples are completely exhausted during diagnostic procedures limiting further additional studies. In this regard, the development of TCR-based biomarkers in liquid biopsy may be an option in advanced NSCLC. Nonetheless, results from limited reports focused on the characterization of peripheral blood TCR repertoire and response prediction are variable, perhaps due to the small sample size, differences in tumor types, sequencing methods or the type of ICB used [25,27,37,40]. Hence, in this study we comprehensively analyzed the TCR-β repertoire in PBMC obtained at two different time points (PRE and FR) from a cohort of 33 advanced NSCLC patients treated in first-line with pembrolizumab, an anti-PD-1 blocker, in monotherapy or in combination with chemotherapy.

We characterized the baseline TCR-β repertoire and found that the number of circulating TCR-β clones significantly decreased in smokers when compared to non-smokers. Although smokers have, in general, a higher TMB, which may further improve the ICB efficacy [14], non-smokers still have longer OS than smokers [41]. Hernandez et al. have demonstrated that smoking can inhibit T cell proliferation by inducing apoptosis, linked to reactive oxygen and nitrogen species [42]. Our results further confirm the adverse effect of smoking on the immune system.

Here, we report that an increase in the TCR-β repertoire richness was associated with better clinical outcomes, suggesting that the increase in the number of T cell clones in peripheral blood may reflect a pharmacodynamic effect of anti-PD-1 antibody therapy. In agreement with our findings, previous studies in melanoma demonstrated that ICBs therapy increased the peripheral blood TCR-β amplitude as reflected in the number of unique TCR clones by high-throughput sequencing in TCR-β chain from PBMCs samples [34,43]; as a consequence, this amplified repertoire may offer more opportunities to recognize tumor neoantigens, leading to improved therapeutic responses.

Our study also revealed that a similar distribution of TCR-β clones between PRE and FR samples was associated with DCB and better PFS. We hypothesized that the pre-existing anti-tumor T cell populations should be maintained during ICB therapy to control tumor progression. In addition to the maintenance of these T cell populations, the enlarged T cell repertoire during ICB therapy could result in the activation of additional neoantigen-specific T cell clones, rendering these patients more likely to respond to immune-based therapies. Similarly, a study in NSCLC patients treated with anti-PD-1/PD-L1 by sequencing peripheral PD-1+ CD8+ T cells TCR-β CDR3 region with NGS showed that higher repertoire similarities between pretreatment and 4–6 weeks post-treatment samples were significantly associated with better clinical outcomes [40]. Additionally, Liu et al. demonstrated that higher similarities in peripheral TCR clones before and after conventional anti-cancer treatments (such as chemotherapy, radiotherapy, targeted therapy, etc.) were associated with better clinical results through profiling the peripheral blood TCR-β CDR3 repertoire via NGS in advanced NSCLC patients [44]. It is generally considered that PD-1 plays a crucial role in regulating immune tolerance and PD-1 blockade therapy can reinvigorate pre-existing TILs that are reactive to neoantigens on tumor cells [45]. However, as discussed by Yost et al., there is increasing evidence that the immune response to anti-PD-1 may originate beyond the tumor microenvironment and rely on peripheral T cell recruitment [46]. Our findings support this argument, making an important contribution to cancer immunotherapy.

The diversified TCR repertoire is generated through somatic recombination of the VDJ gene segments. In our study, high usage of TRBV6-5 was associated with disease progression and a poor prognosis. One possible explanation for the negative prognostic role of TRBV6-5 is that the T-cells containing this segment may not be activated by tumor-associated antigens, and cannot recognize neoantigens. This hypothesis agreed with a study concerning deep TCR-β sequencing in isolated T cells from tumor tissue of patients with prostate cancer, in which TRBV6-5 was specific to the adjacent tumor samples and could not be detected in the tumor tissue [47].

Finally, our data revealed that TRBV20-1 was the most abundant V segment in our cohort. Moreover, the presence of higher frequencies of these TCR clones in peripheral blood predicts survival to anti-PD-1 therapy. There is limited information regarding the prognostic or predictive value of V and J-gene usage in circulating T cells in this clinical context, and to our knowledge, none has been found in NSCLC to date. In line with our results, TRBV20-1 has been reported previously as one of the most used V-gene in tumor-infiltrating T lymphocytes in several solid tumors [48]. Particularly, in lung cancer, Wang et al. demonstrated that the TRBV20-1 usage was higher in tumor compared with normal lung tissues by sequencing the TCR-β CDR3 region [24]. These data align with our results suggesting that the T cell clones containing TRBV20-1 may recognize tumor neoantigens, and should be maintained when undergoing ICB treatment to control the growth of tumor cells. Thus, we propose that analysis of circulating TRBV20-1 usage may be predictive of increased survival in anti-PD-1 treated NSCLC patients.

Although there are some limitations in this study, mainly related to the sample size, which could limit the generalization of our findings, and the lack of an external validation set, the results presented here support that characterization of blood TCR repertoire provides valuable clinical information using a minimally invasive approach. Moreover, analysis and comparison of paired blood and tumor tissue samples should be performed in subsequent extensive cohorts, in order to identify accurately the functional anti-tumor T cells. Besides, the correlation and possible combination with other validated biomarkers, such as TILs, PD-L1 expression on tumor-infiltrating immune cells and TMB, should also be analyzed to provide a better prognostic signature of immunotherapy in lung cancer. Nevertheless, the analyses presented here demonstrate the feasibility and clinical relevance of sequencing the CDR3 region of TCR-β chain to characterize the circulating T cell repertoire, enhanced by the decrease of sequencing costs and the easy access to blood samples rather than tumor tissue samples.

## 5. Conclusions

Altogether, our results show that TCR-β based biomarkers are useful, not only to assess the immune response, but also to predict the clinical outcomes for immunotherapy in NSCLC patients. Furthermore, the TRBV20-1 usage might be applied as a novel biomarker, facilitating patient selection, in NSCLC patients treated with pembrolizumab.

## Figures and Tables

**Table 1:** Clinicopathological characteristics of the patients included in the study.

Clinicopathological Characteristics	*n* = 33	%
**Age** (years)(median, range)	65 (46–87)	
**Sex**		
Male	24	72.7
Female	9	27.3
**Smoking status**		
Smoker	25	75.8
Former smoker	6	18.2
Never smoker	2	6.1
**Histological subtype**		
Adenocarcinoma	24	72.7
Squamous carcinoma	4	12.1
Large cell carcinoma	1	3.0
Poorly differentiated	4	12.1
**Stage classification by TNM** ^1^ **standard**		
IIIA ^2^	2	6.1
IIIB	3	9.1
IIIC	1	3.0
IVA	16	48.5
IVB	11	33.3
**PD-L1 TPS** ^3^		
<1%	6	18.2
1-49%	3	9.1
≥50%	24	72.7
**First-line treatment**		
Pembrolizumab monotherapy	24	72.7
Pembrolizumab-Cisplatin-Pemetrexed	9	27.3

^1^ TNM: tumor–node–metastasis staging. ^2^ IIIA: patients not candidates for surgery, radiation, or chemotherapy. ^3^ PD-L1 expression was assessed by tumor proportion scores (TPS) and reported by using a three cut-point system: TPS < 1%, TPS 1%–49%, and TPS ≥ 50%.

## Data Availability

The data presented in this study are available in this article and attached supplementary files.

## Supplementary Materials

The following are available online at https://www.mdpi.com/article/10.3390/cancers13122950/s1, Figure S1: Distribution of reads obtained by TCR-β sequencing, Figure S2: Correlation between peripheral TCR-β repertoire features and response to anti-PD-1 in pretreated NSCLC patients, Figure S3: Kaplan–Meier survival curves according to baseline TCR-β features in pretreated NSCLC patients, Figure S4: Correlations between changes in absolute lymphocyte count (ALC) and changes in TCR richness in anti-PD-1 treated NSCLC patients, Figure S5: Correlation of dynamic evenness and convergence of TCR-β repertoire in peripheral blood with response in anti-PD-1 treated NSCLC patients, Figure S6: Kaplan–Meier survival curves according to TCR-β evenness and convergence dynamics in anti-PD-1 treated NSCLC patients, Figure S7: Correlation of TCR-β clones similarity before and during the anti-PD-1 treatment with response in NSCLC patients, Figure S8: Kaplan–Meier overall survival (OS) curves according to the Jaccard similarity index, Figure S9: Usage of V and J-gene segments in pretreatment and first response assessment samples, Table S1: Detailed clinicopathological characteristics of the patients included in the study, Table S2: Sample characteristics and TCR-β CDR3 sequencing information of the patients included in the study, Table S3: Comparison of TCR-β repertoire characteristics between PRE and FR samples, Table S4: Correlation of basal TCR-β repertoire characteristics with clinicopathological variables in pretreated NSCLC patients, Table S5: Correlation of TCR-β repertoire characteristics with response in NSCLC patients.
