# Supplementary material for: Characterization of Circulating T Cell Receptor Repertoire Provides Information about Clinical Outcome after PD-1 Blockade in Advanced Non-Small Cell Lung Cancer Patients
Source: Cancers (Basel). 2021 Jun 12;13(12):2950. doi: 10.3390/cancers13122950 (PMC8231221; doi:10.3390/cancers13122950)
Supplement: Supplementary file 1 [file cancers-13-02950-s001.zip › cancers-1210306-suppl.pdf]

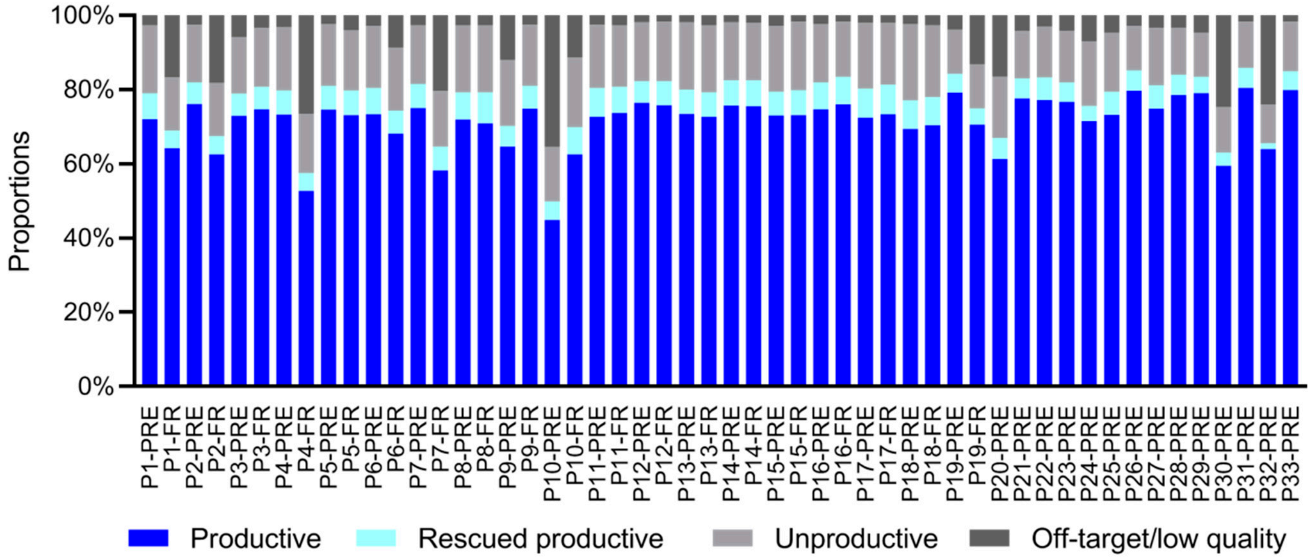

Figure S1. Distribution of reads obtained by TCR-β sequencing. After the first stage of data processing, raw sequencing reads were classified in: i) "productive" (in blue), reads having an in-frame variable and joining gene, and no stop codons; ii) "rescued productive" (in light blue), reads having an in-frame variable and joining gene, and no stop codons after INDEL error correction; iii) "unproductive" (in light gray), reads that had uncorrectable sequencing or PCR errors, which led the rearrangement to have out-of-frame variable and joining genes or a premature stop codon; iv) "off-target/low quality" (in gray), reads that were of low quality or represented the product of an off-target amplification. Productive and rescued productive reads were considered as total productive reads for further analysis process. The library P10-PRE presented the lowest proportion of total productive reads (50%), which could be associated with RIN = 7, indicating inferior integrity of the RNA molecule, but within the acceptable range. The other samples presented a proportion of productive plus rescued productive reads above 50%. P: patient; PRE: pretreatment samples; FR: first response assessment samples.

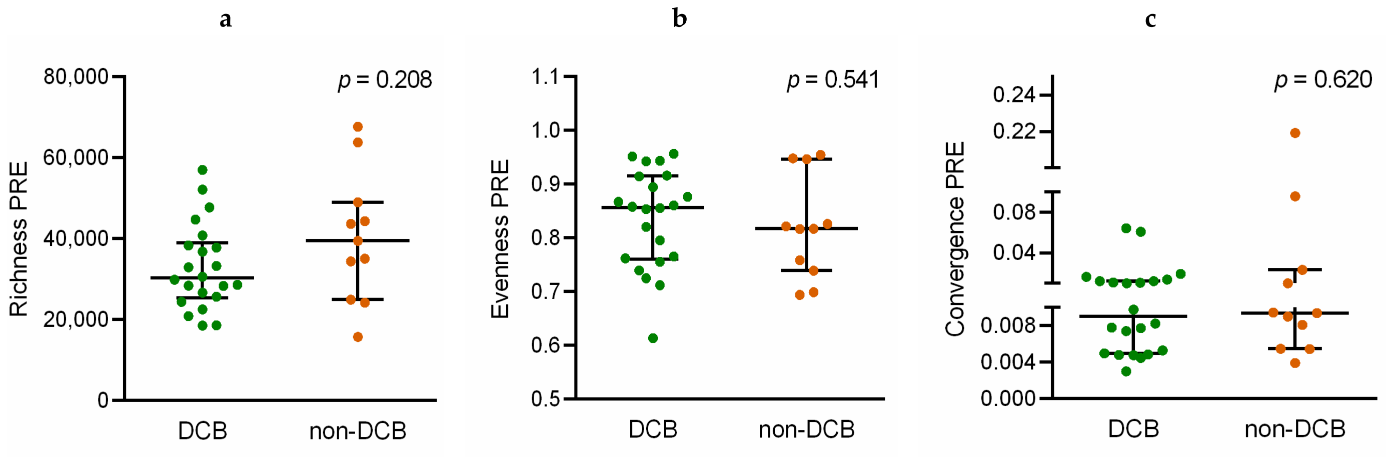

Figure S2. Correlation between peripheral TCR-β repertoire features and response to anti-PD-1 in pretreated NSCLC patients. Comparison of pretreatment richness (**a**), evenness (**b**), and convergence (**c**) in patients with durable clinical benefit (DCB; in green) vs. non-durable clinical benefit (non-DCB; in orange). *P*-values were obtained using the Mann–Whitney test. Error bars represent the interquartile range with a line at the median. Each dot represents a patient. PRE: pretreatment.

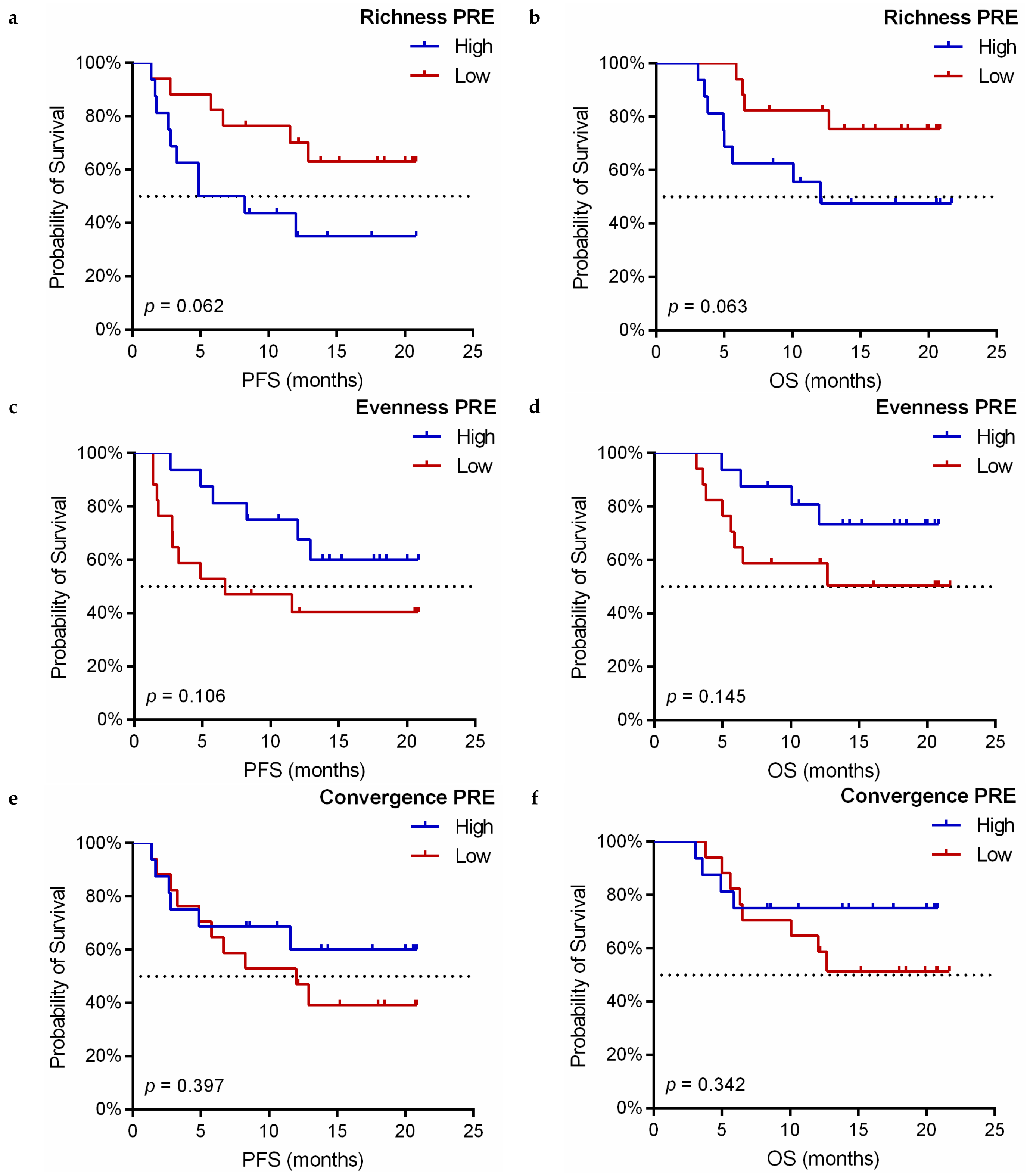

Figure S3. Kaplan–Meier survival curves according to baseline TCR-β features in pretreated NSCLC patients. (**a,b)** Progression-free survival (PFS) and overall survival (OS) curves in terms of pretreatment richness. **(c,d)** PFS and OS curves in terms of pretreatment evenness. **(e,f)** PFS and OS curves in terms of pretreatment convergence. The medians of each index (richness, median = 33,284; evenness, median = 0.8263; convergence, median = 0.0094) were used as cutoff values to divide patients into high and low groups. *P*-values were obtained using the log-rank test. PRE: pretreatment.

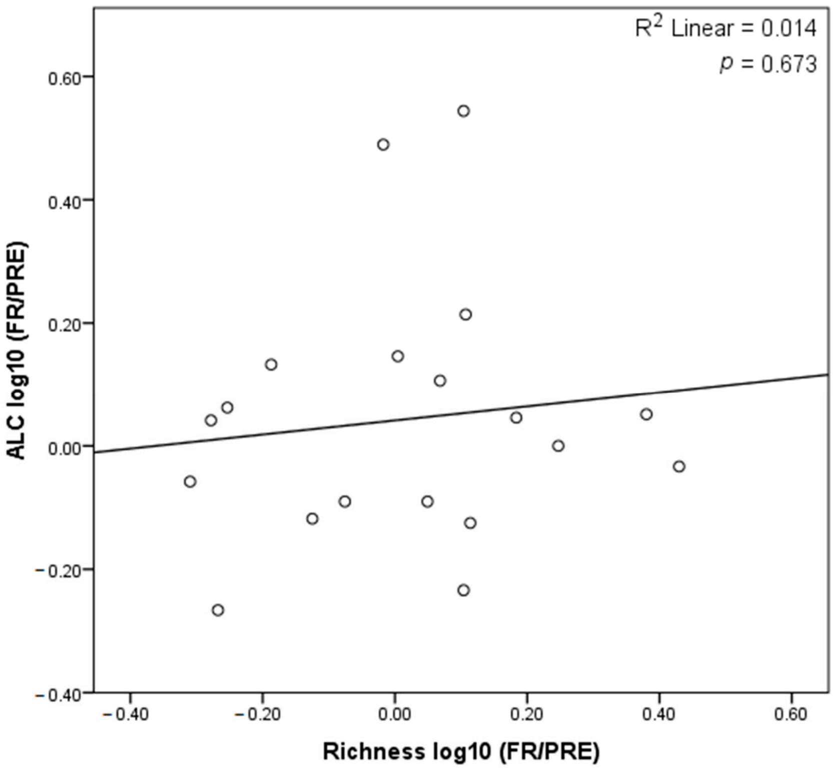

Figure S4. Correlations between changes in absolute lymphocyte count (ALC) and changes in TCR richness in anti-PD-1 treated NSCLC patients. Statistical analysis was performed using the Spearman's rank test. The X-axis corresponds to the ALC log_{10} (FR/PRE), and Y-axis corresponds to the richness log_{10} (FR/PRE) between pretreatment (PRE) and first response assessment (FR). Each dot represents a patient.

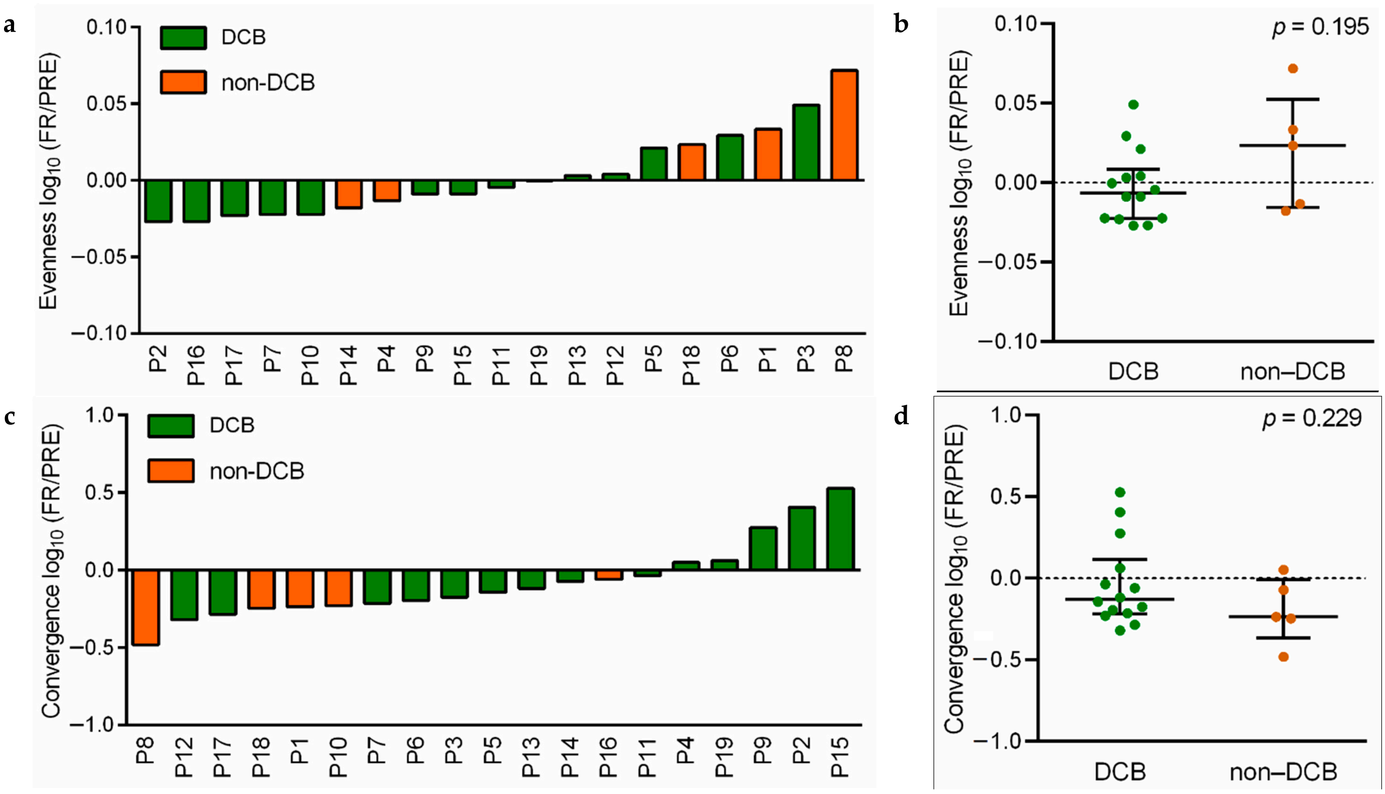

Figure S5. Correlation of dynamic evenness and convergence of TCR-β repertoire in peripheral blood with response in anti-PD-1 treated NSCLC patients. **(a,c)** Evenness (a) and convergence (c) $\log_{10}$ (FR/PRE) between pretreatment (PRE) and first response assessment (FR) in patients with durable clinical benefit (DCB; in green) and non-durable clinical benefit (non-DCB; in orange). Each column represents a patient. **(b,d)** Comparison of evenness (b) and convergence (d) $\log_{10}$ (FR/PRE) between patients with DCB and non-DCB. *P*-values were obtained using the Mann–Whitney test. Error bars represent the interquartile range with a line at the median. Each dot represents a patient.

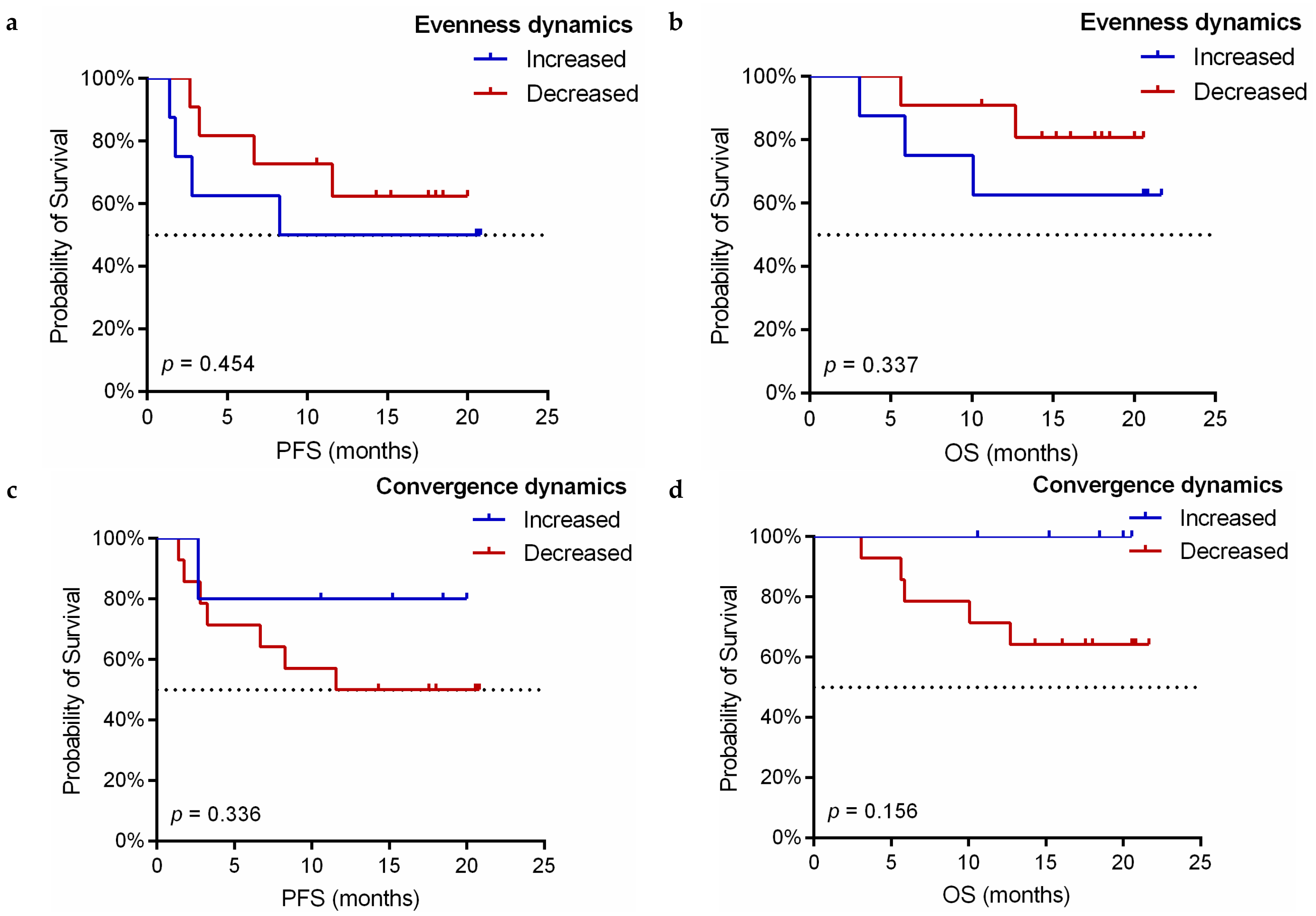

Figure S6. Kaplan–Meier survival curves according to TCR-β evenness and convergence dynamics in anti-PD-1 treated NSCLC patients. **(a,b)** Progression-free survival (PFS) and overall survival (OS) curves stratified in increased ($n = 8$) vs. decreased evenness ($n = 11$). **(c,d)** PFS and OS curves stratified in increased ($n = 5$) vs. decreased evenness ($n = 14$). *P*-values were obtained using the log-rank test.

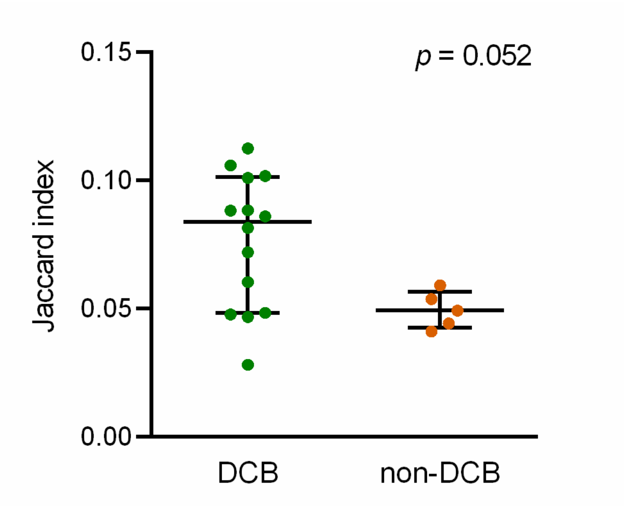

Figure S7. Correlation of TCR-β clones similarity before and during the anti-PD-1 treatment with response in NSCLC patients. *P*-value was obtained using the Mann–Whitney test. Error bars represent the interquartile range with a line at the median. Each dot represents a patient. DCB: durable clinical benefit, in green; non-DCB: non-durable clinical benefit, in orange.

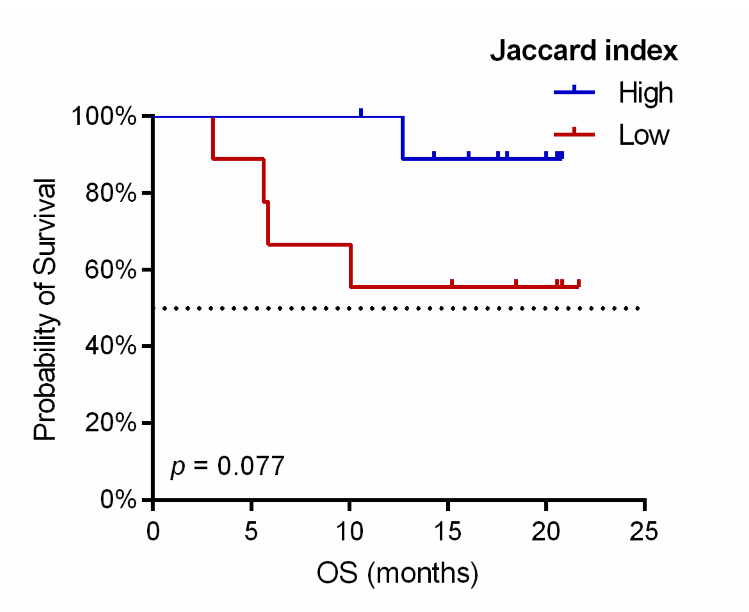

Figure S8. Kaplan–Meier overall survival (OS) curves according to the Jaccard similarity index. Jaccard similarity index was calculated as the total number of shared clones divided by the total number of unique clones across PRE, and FR samples of the same patient and the median = 0.0605 was used as the cutoff value to define high (blue line) and low (red line) groups. *P*-value was obtained using the log-rank test.

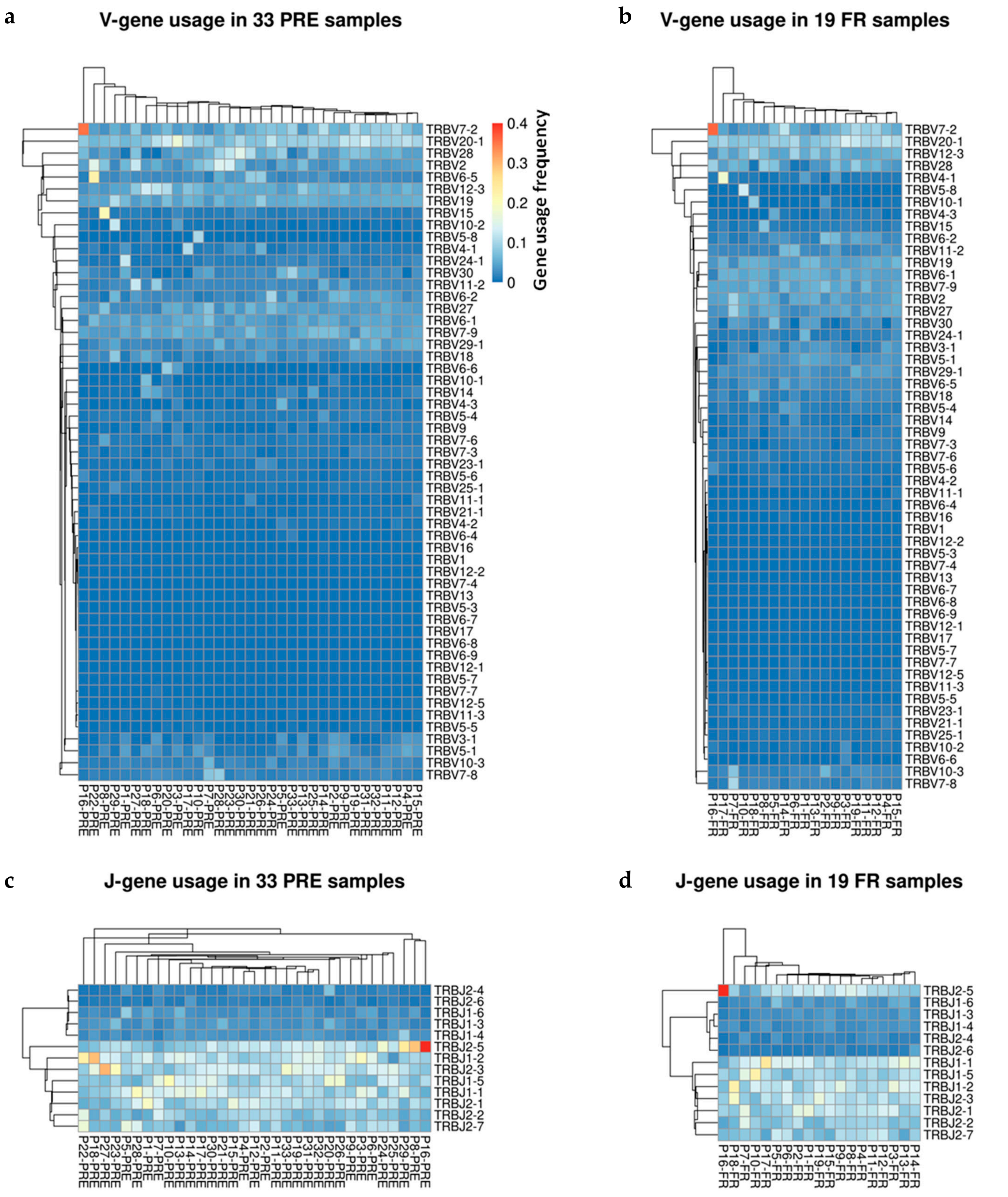

Figure S9. Usage of V and J-gene segments in pretreatment and first response assessment samples. **(a)** V-gene usage frequency in 33 PRE samples. **(b)** V-gene usage frequency in 19 FR samples. **(c)** J-gene usage frequency in 33 PRE samples. **(d)** J-gene usage frequency in 19 FR samples. The color bars indicate the gene usage frequency of each sample. PRE: pretreatment. FR: first response assessment.
